# Dynamic Analysis and Vibration Control of Additively Manufactured Thin-Walled Polylactic Acid Polymer (PLAP) and PLAP Composite Beam Structures: Numerical Investigation and Experimental Validation

**DOI:** 10.3390/ma17225478

**Published:** 2024-11-09

**Authors:** Ali Raza, Magdalena Mieloszyk, Rūta Rimašauskienė, Vytautas Jūrėnas

**Affiliations:** 1 Faculty of Mechanical Engineering and Design, Kaunas University of Technology, Studentų Str. 56, LT-51424 Kaunas, Lithuania; 2 Institute of Fluid-Flow Machinery, Polish Academy of Sciences, Fiszera 14 Str., 80-231 Gdansk, Poland; mmieloszyk@imp.gda.pl; 3 Institute of Mechatronics, Kaunas University of Technology, Studentų Str. 56, LT-51424 Kaunas, Lithuania

**Keywords:** thin-walled beam, MFC, modal frequencies, THz spectroscopy, vibration control, amplitude spectrum

## Abstract

This study primarily presents a numerical investigation of the dynamic behavior and vibration control in thin-walled, additively manufactured (AM) beam structures, validated through experimental results. Vibration control in thin-walled structures has gained significant attention recently because vibrations can severely affect structural integrity. Therefore, it is necessary to minimize these vibrations or keep them within acceptable limits to ensure the structure’s integrity. In this study, the AM beam structures were made of polylactic acid polymer (PLAP), short carbon fiber reinforced in PLAP (SCFR|PLAP), and continuous carbon fiber reinforced in PLAP (CCFR|PLAP), with 0°|0° layer orientations. The finite element modeling (FEM) of the AM beam structures integrated with macro fiber composite (MFC) was carried out in Abaqus. The initial four modal frequencies of bending modes (BMs) and their respective modal shapes were acquired through numerical simulation. It is crucial to highlight the numerical findings that reveal discrepancies in the 1st modal frequencies of the beams, ranging up to 1.5% compared to their respective experimental values. For the 2nd, 3rd, and 4th modal frequencies, the discrepancies are within 10%. Subsequently, frequency response analysis (FRA) was carried out to observe the frequency-dependent vibration amplitude spectrum at the initial four BM frequencies. Despite discrepancy in the amplitude values between the numerical and experimental datasets, there was consistency in the overall amplitude behavior as frequency varied. THz spectroscopy was performed to identify voids or misalignment errors in the actual beam models. Finally, vibration amplitude control using MFC (M8507-P2) was examined in each kinematically excited numerical beam structure. After applying a counterforce with the MFC, the controlled vibration amplitudes for the PLAP, SCFR|PLAP, and CCFR|PLAP configurations were approximately ±19 µm, ±16 µm, and ±13 µm, respectively. The trend in the controlled amplitudes observed in the numerical findings was consistent with the experimental results. The numerical findings of the study reveal valuable insights for estimating trends related to vibration control in AM beam structures.

## 1. Introduction

Currently, vibration suppression in structural dynamics has emerged as a prominent domain of research. These structures, either with simple or intricate designs, are susceptible to vibrations caused by various sources such as machinery noise, wind loads, human activities, and others [1]. These vibrations can significantly compromise structural integrity, leading to premature fatigue and eventual failure. Therefore, it is necessary to mitigate these vibrations or keep them within an acceptable limit to minimize the risk of structural failure [2,3]. It is noteworthy to describe that thin-walled composite beam structures have a considerable number of engineering applications. Structures such as helicopter blades, aircraft blades, robotic arms, [4] and wing turbine blades demonstrate the geometry of thin-walled beams. However, flutter is a challenging issue concerned with these kinds of structures and needs to be minimized or eliminated [5]. In recent times, structures are manufactured using various types of composite materials to obtain high specific stiffness as well as specific strength. On the other hand, vibration suppression has emerged as a crucial issue for the operational performance of the system due to the flexible properties of the composite structures [6,7].

Many vibration suppression technologies, such as active control, semi-active control, passive control, and integrative control, have been implemented recently to enhance system integrity. A passive control scheme includes incorporating various damping materials or devices (e.g., mass, spring, and damper), producing the control counter force in reaction to external disturbances and does not need any external electrical source for operation. However, other schemes generally require actuators, sensors, and controllers to function. These can provide control counter forces to the system based on the real-time data but need external electrical sources to activate these devices [8]. It is important to highlight that active control schemes are increasingly used in numerous engineering fields due to the easy market accessibility of a range of smart materials, particularly piezoelectric [9,10]. Piezoelectric materials are often used for energy harvesting [11], monitoring structural integrity [12], and vibration suppression applications [13], due to their effective electromechanical capabilities, substantial blocking force, excellent stiffness, and prompt response [14]. Piezoelectric materials, such as lead zirconium titanate (PZT), are commonly employed in numerous industrial applications due to their excellent stiffness and ability to generate high actuation force. Additionally, they may be implemented as sensors and actuators attributed to direct piezoelectric and converse phenomena, respectively. However, they have some constraints, such as brittle behavior, inadequate flexibility, and limited capability to adapt curved surfaces [2]. The polyvinylidene fluoride is an alternative piezoelectric material that is relatively more flexible than PZT but generates less actuation force.

To alleviate the challenges of piezoelectric materials for real-life application, thorough studies have been carried out on the polymeric matrix reinforced with piezoceramic fibers [15,16]. In 1999, NASA developed various types of macro fiber composites (MFCs) to overcome their limitations. The polymeric matrix was reinforced with rectangular-shaped piezoceramic fibers and positioned within protective and electrode wraps [17,18,19]. Afterwards, in 2002, the Smart Materials Corp. commenced the production and supply of the MFCs under a licensed manufacturer agreement for NASA-developed technology [20].

Dafang et al. carried out an experimental study on active vibration control of a flexible beam manufactured from epoxy resin and fiberglass, employing the independent mode control (IMC) technique to control the first three modes independently. Piezoelectric patches, used as actuators, were integrated with the beam at designated positions to damp vibrations. The findings demonstrate that the modal damping of the beam structures was significantly enhanced by using the IMC technique. To perform the numerical simulation, the dynamic equation governing the beam was formulated using Hamilton’s principle. The simulation results were closely aligned with the experimental results. The findings from experiments and simulations illustrate that employing the piezoelectric actuators enabled the IMC technique to effectively suppress vibrations [21]. Raza et al. conducted numerical simulations on the vibration control of beam structures with distinct material properties integrated with MFC (M8507-P2) using the ANSYS R19.1 software. Modal analysis was thoroughly performed on each beam to assess the modal frequencies and modal shapes. Additionally, vibration control employing MFC was executed on each beam, with the maximum vibration reduction noted in the beam with the highest 1st resonant frequency. The findings confirm that this scheme could be effectively implemented across various materials to control vibration [22]. Silva et al. conducted an experimental study on active vibration control in composite beams by applying electromagnetic actuators. A fuzzy logic methodology was employed to control the vibration amplitude of the beams. An identical mathematical model of the experimental setup was developed using the unique methodology, and numerical simulations were performed on the model using the MATLAB platform. Both numerical and experimental results for vibration suppression of the first mode in frequency and time aspects were compared and validated. The insights reveal the robustness of the control methodology applied to suppress vibration amplitude [23]. Rimašauskienė et al. accomplished an experimental investigation to evaluate the combined impact of a unique active–passive vibration control methodology on the beam structure with the application of an MFC patch. The results demonstrate that the suggested methodology effectively facilitates in the vibration suppression of the beam structure [7]. Raza et al. performed a comprehensive investigation on the dynamic characteristics of 3D-printed beam structures integrated with MFC (M8507-P2). The beams were developed using polylactic acid polymer (PLAP), short carbon fiber reinforced in PLAP (SCFR|PLAP), and continuous carbon fiber reinforced in PLAP (CCFR|PLAP) beam structures oriented at two distinct layer patterns: 0°|0° and 0°|90°. The deformation, decrement coefficient, and modal characteristics were assessed, and the impact test was conducted on each beam to observe the influence of distinct layer patterns on dynamic characteristics. The findings demonstrate that the two distinct orientations (0°|0° and 0°|90°) have a significant effect on the dynamic characteristics of the beam [24]. Moreover, Raza et al. presented a detailed study of vibration damping with the influence of MFCs in 3D-printed composite structures made of CCFR|PLAP and continuous glass fiber reinforced in PLAP (CGFR|PLAP) with two distinct orientations (0°|0° and 0°|90°). The findings illustrate that the structures with 0°|0° orientation exhibit higher vibration damping compared to those with the 0°|0° orientation [25]. Karimi et al. presented a comprehensive review of the fused deposition modeling (FDM) approaches employed in the 3D printing of continuous and short fiber-reinforced composites. Continuous fiber-reinforced composite parts are receiving greater interest in industries, compared to short fibers, due to their excellent strength. Moreover, various fiber reinforcement composite fabrication approaches were discussed, especially two approaches: in situ fusion and ex situ prepreg. However, voids and irregularities were found in the parts fabricated by these two approaches. To alleviate these problems, new approaches have been established by researchers through modifications to these two techniques, resulting in the filling of voids and irregularities and enhancing the mechanical properties of composites parts [26]. Wani et al. conducted a comprehensive review of various control methodologies, including active, passive, and hybrid control, for effectively controlling vibrations in structures. The adoption of a specific control technique is influenced by the complexity of the structural system. Furthermore, it was reported that more than one type of technique may be implemented to control vibrations effectively [27]. Ding et al. presented a novel vibration control methodology to manage vertical vibrations within the engineering framework. Traditional tuned fluid column dampers (TFCDs) are employed to mitigate vibrations in horizontal orientations. This study introduces a novel methodology based on TFCD, aimed at successfully suppressing vibrations in vertical orientations. Afterward, the motion equations of the proposed system were formulated using Lagrange equations and further validated through numerical simulations in the ANSYS suite. The outcomes provide valuable insights into the capabilities of the suggested TFCD methodology and illustrate its potential to suppress vibrations in the vertical direction [28]. Wang et al. proposed a practical technique, ‘dynamic mode decomposition (DMD) approach’, to formulate a low-order state-space model (SSM) for mitigating vibrations in a cable strut-based framework, relying on data regarding the framework’s behavior and input control force. The data related to the input control force were determined by stimulating the framework with integrated actuators. A novel linear quadratic regulator (LQR)-based approach, DMD-LQR, was developed using the DMD-based SSM. This approach has been proven to be more computationally effective relative to the FEM-derived LQR approach. The efficacy of the novel approach (DMD-LQR) was also demonstrated through numerical simulations. The findings illustrate the effectiveness of this novel approach in mitigating vibrations [29]. Zang et al. conducted analytical and experimental studies on the nonlinear vibration control in coupled carbon composite beam structures with secured ends under thermal influence. The modal characterization and mode frequencies of the coupled beams were determined using the ‘Rayleigh–Ritz’ approach and confirmed by FEM. Dynamic equations of motion were formulated using Lagrange’s set of equations and the ‘Galerkin’ approach. Additionally, the frequency-dependent amplitude response was assessed through the harmonic balance technique and verified through the ‘Runge–Kutta’ approach. For the experimental study based on the analytical study, NiTiNOL-steel wires were placed on the coupled beams in accordance with a predefined layout. The experimental findings demonstrate that the steel wires effectively damp the vibrations in the coupled composite beams and align with the analytical results [30]. Guang Sun et al. presented a ‘linear active disturbance rejection control (LADRC)’ approach to mitigate vibrations in beam structures integrated with piezoelectric patches acting as actuators. The beam structures were externally stimulated by harmonic loads. Initially, the fundamental equations of the beam with actuators were formulated utilizing the Euler–Bernoulli beam model along with Lagrange’s equations. The LADRC approach for the beam with piezoelectric patches was derived from these fundamental equations. To confirm the accuracy of the analytical study, the same analysis was performed using ANSYS and experimentally. The findings validate that the applied approach has a substantial effect on controlling vibrations in beam structures [31]. Soltani et al. carried out a thorough investigation of vibration control of multi-layer sandwich-based micro-beams manufactured from piezoelectric materials. To examine the effectiveness of control measures for the sandwich beam structure and to assess the relevant mechanical characterization, both Hamiltonian and ‘generalized differential quadrature (GDQ)’ methodologies were applied in determining the solution discretely. Moreover, the authors designed several controllers to investigate the tracking characterization and vibration dampening of the system. The findings of this investigation demonstrated that the Linear Quadratic Integral-based control technique performed considerably more effectively in controlling vibration and enhancing tracking characteristics [32]. Moreira et al. proposed a unique passive damping approach for light and elastic frameworks by introducing viscoelastic material (VM) to address vibration challenges and potential early failure under dynamic loading conditions. They incorporated indentations on the internal surfaces of the host structure (bottom layer), along with the confining layer (upper layer) sandwiching the VM layer, which adopted a wave-like configuration. The modeling of this sandwich structure was performed using the ‘NX Nastran software’ in association with the Siemens FEMAP software, with the facilitation of the MATLAB platform for mesh creation. The insights confirm that the VM layer significantly enhances passive damping treatment [33]. Kamel et al. developed a dynamic model of a carbon composite-based cantilever beam in ANSYS using FEM to control vibrations. Frequency analysis of the dynamic model was conducted in ANSYS considering four distinct scenarios. The state space model (SSM) of the smart beam for each scenario was created in the MATLAB platform using the results obtained from ANSYS. Subsequently, a PID controller was developed using the SSM derived from the first scenario in the MATLAB platform and confirmed against the other three scenarios. To enhance the performance of the system, three additional smart controllers based on fuzzy logic were studied. The findings reveal that fuzzy-based controllers contributed a significant role in the vibration control of the beam system [34].

This numerical study aims to validate previously published experimental investigations [35]. Prior research extensively explored the dynamic characteristics of kinematically stimulated, additively manufactured (AM) beam structures, including modal characterization, frequency-dependent amplitude spectra, and vibration control influenced by MFC (M8507-P2). The beam structures were fabricated using PLAP, SCFR|PLAP, and CCFR|PLAP with 0°|0° layer orientations, and their fabrication process has been thoroughly discussed in a published experimental work [35].

This is the first time the dynamic characteristics and vibration control in numerical models of PLAP, SCFR|PLAP, and CCFR|PLAP with [0°|0°] layer sequences integrated with MFC (M8507-P2) have been comprehensively discussed and validated against previously published experimental results. However, challenges arose due to the general assumptions adopted in the numerical simulations compared to the experimental scenarios.

Initially, the finite element modeling (FEM) of PLAP, SCFR|PLAP, and CCFR|PLAP with MFC (M8507-P2) was performed in the Abaqus CAE 2024 platform. Following this, the modal characterization assessment of each numerical beam model was conducted to assess the modal frequencies and their respective modal shapes. The discrepancies in numerical and experimental modal frequencies for each beam have been reported separately. Additionally, frequency response analysis (FRA) was carried out to assess the frequency-dependent amplitude spectra of the numerical models, and comparisons were made with the experimental results. In the next section, THz spectroscopy was performed to reveal voids or misalignment errors in the 3D-printed layers of actual beam models. Finally, vibration control in each numerical model, utilizing MFC counterforces, was thoroughly examined and compared with experimental results. A detailed investigation of the discrepancies between the simulation and experimental results has also been presented. Based on the findings, the beam structure that exhibited the highest vibration suppression was identified.

## 2. Materials and Methodology

**Figure 1:**
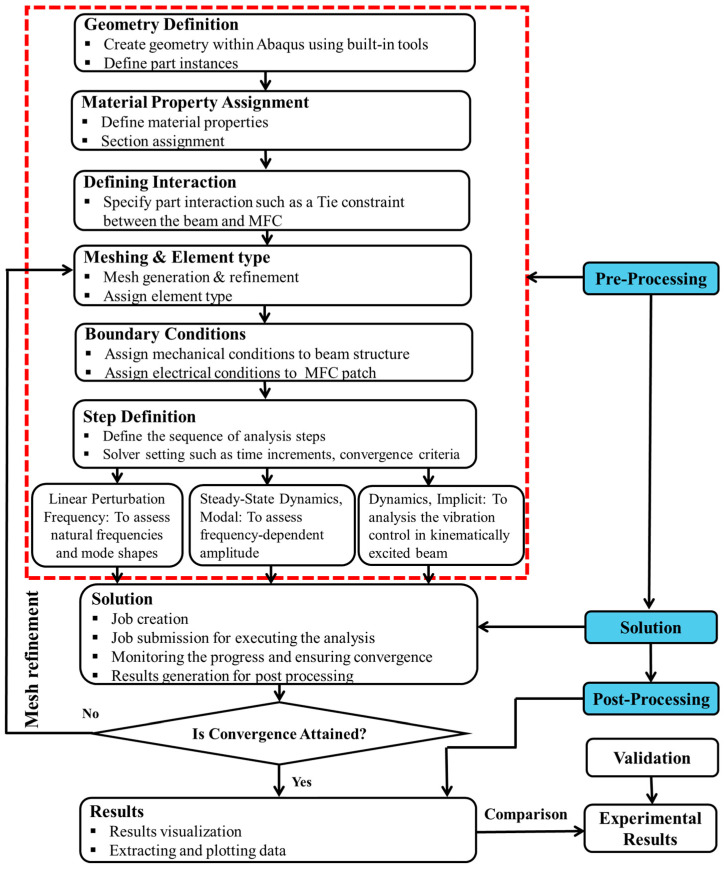
Overview of steps involved in numerical simulation.

In this numerical simulation investigation, the 0°|0°-oriented PLAP, SCFR|PLAP and CCFR|PLAP beam structures were modeled to study dynamic characteristics such as modal characterization assessment and FRA, with the goal of mitigating vibrations in beam structures integrated with MFCs by implementing open-loop active vibration control (OL-AVC). The numerical results were thoroughly compared and validated with previously published experimental study by the authors. The overview of the steps involved in the numerical simulation investigation has been demonstrated in Figure 1.

In Figure 1, three main steps involved in the numerical simulation are illustrated: pre-processing, solution, and post-processing. In the pre-processing step, the geometry of the beam structure with the MFC is created and defined. The material properties are assigned to the beam and MFC separately, interaction is established between them, element types are assigned, and meshing is performed. Then, boundary conditions are applied, followed by defining various analysis steps such as linear perturbation frequency (to assess natural frequencies and mode shapes), steady-state dynamics (to assess frequency-dependent amplitude), and dynamics implicit (to analyze vibration control with MFCs).

In the solution step, a job is created for each step and executed for analysis. The progress of each numerical simulation is monitored, and the convergence of the results is confirmed. If the results do not converge, mesh refinement is performed, and the analysis steps are repeated.

In the post-processing step, the results are visualized and examined, and the required data are extracted. The graphs are generated to describe the results. Finally, the simulation results are validated against the experimental results to confirm the accuracy of the numerical findings.

A thorough discussion of numerical modeling, simulation investigation, and validation with the experiment are discussed in subsequent sections.

### 2.1. Finite Element Modeling (FEM)

**Figure 2:**
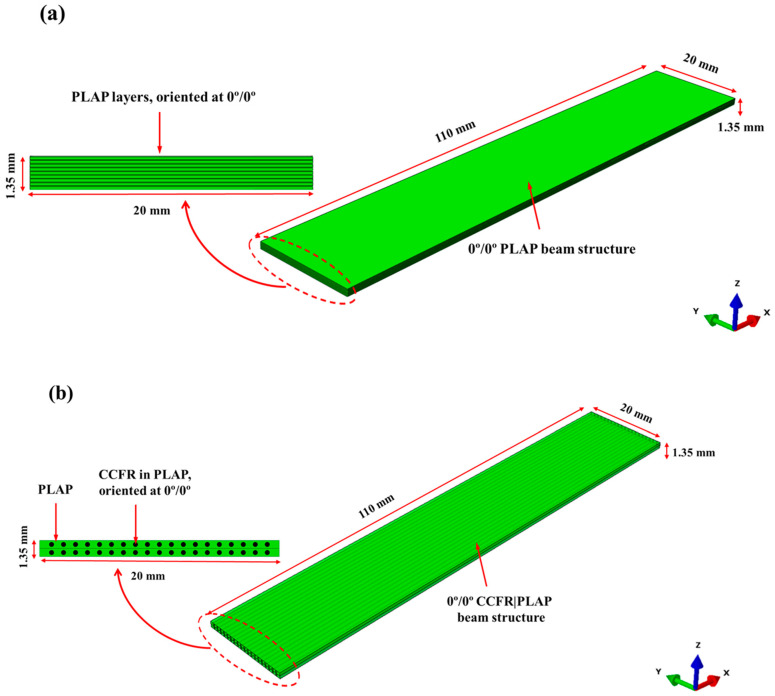
Modeling beam structures oriented at 0°|0°: (**a**) approach for PLAP and SCFR|PLAP beam structures and (**b**) approach for CCFR|PLAP.

**Figure 3:**
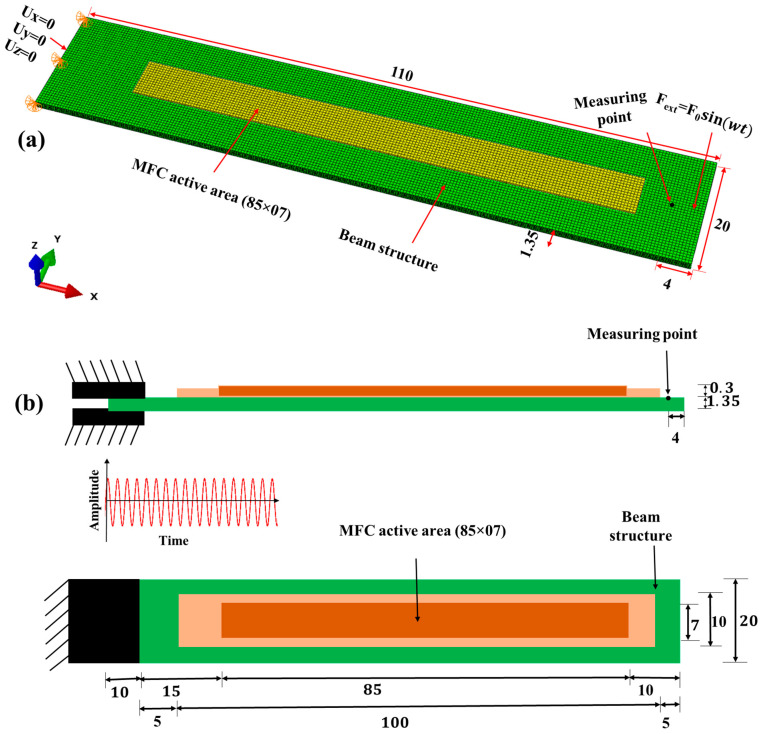
Beam structures integrated with MFC, illustrating boundary conditions (units in mm): (**a**) FEM model and (**b**) schematic view of experimental sample.

In this section, the FEM of laminated beam structures (length: 110 mm; width: 20 mm; thickness: 1.35 mm) with MFC patches is outlined. The overall effective dimensions of both numerical models and AM beam structures were considered similar. However, the actual length of the AM beam was 120 mm, as a 10 mm portion of the beam was placed within a support to firmly fix the beam from one side. Thus, the effective length of AM beams during the experiments was used 110 mm. Firstly, the PLAP and SCFR|PLAP laminated beams with [0°|0°] layer sequences were individually modeled as continuum solid shells (CSS8). The layup-ply approach was adopted to describe the layer arrangement. The [0°|0°] layer sequence indicates that all layers were aligned parallel (along the same axis) to each other without any angular deviation between them, as presented in Figure 2a. Conversely, the CCFR|PLAP beam composite with [0°|0°] fiber layer sequences, was represented as continuum solid shells (CSS8) using a representative volume element (RVE) with an ~18% volume fraction of fibers (consistent with AM beam structures). Similarly, in the CCFR|PLAP model, [0°|0°] fiber layer sequences showed that the direction of the fibers in both layers was parallel to each other (along the same axis), as illustrated in Figure 2b. The material parameters for modeling beam structures are detailed in Table 1. Subsequently, the FEM of integrated MFC (M8507-P2) was performed using piezoelectric solid elements (C3D20RE). The active part of the MFC (length: 85 mm; width: 0.7 mm; thickness: 0.3 mm) was simplified as a homogeneous material rather than an intricate design. The material parameters of the MFC are listed in Table 2. The active portion of the MFC was affixed to each beam structure in accordance with AM samples, i.e., 15 mm from the fixed end. The Tie constraint was implemented to ensure interaction between the surfaces of the beam and the MFC. After numerically modeling the geometry, hexahedral (hex) meshing with refinement was performed on the MFC and each beam structure, resulting in no additional significant changes in the computed natural frequencies. The aim of the mesh refinement was to attain greater exactness; however, excessive refinement prolonged the computational time required to obtain the desired output, rendering it impractical for use. Further, mechanical boundary conditions involved fixing one side of the beam, with nodes restricted to displace in the X, Y, and Z direction, as depicted in Figure 3a. On the other side, a sinusoidal force was employed to kinematically excite the beam. However, due to limitations of the Abaqus, it was not possible to apply the excitation force through the fixed side as was carried out in the experiments exhibited in Figure 3b. The electrical boundary conditions, such as a sinusoidal signal (U_MFC_), were applied from the upper side of the MFC, while the bottom side was grounded (0 V).

### 2.2. Modal Analysis

The Lanczos methodology has been employed to compute the modal natural frequencies and corresponding mode shapes of beam structures without applying external forces. This process involves specifying the geometric configurations of the beam structures, material characteristics, and boundary conditions, such as fixing one side of the beam structure firmly.

The Abaqus employs the following fundamental governing equations to determine the modal frequencies and mode shapes, as explained in the Abaqus documentation [41]:

(1) $$M\ddot{z}(t)+Kz(t)=0$$

The above equation describes the motion of the undamped system with multiple degrees of freedom.

Where M, $\ddot{z}(t)\;,\;z(t)$, K describe mass matrix, acceleration vector, displacement vector, and stiffness matrix.

Assuming harmonic motion of the dynamic system,

(2) $$z(t)=\Phi_{n}\sin(w_{n}t)$$

By substituting Equation (2) into Equation (1),

(3) $$M({-w}_{n}^{2}\Phi_{n}\sin(w_{n}t))+K(\Phi_{n}\sin(w_{n}t))=0$$

This simplifies to the following:

(4) $$(K-w_{n}^{2}M)\Phi_{n}=0$$

Equation (4) represents equilibrium equation governing the dynamics of structure for undergoing free vibration that is formulated as an eigenvalue problem:

(5) $$\lambda_{n}={\;w}_{n}^{2};\;(K-\lambda_{n}M)\Phi_{n}=0$$

where K, $\Phi_{n}$, $\lambda_{n},$ and M represent stiffness matrix, eigenvectors, eigenvalues, and mass matrix, respectively.

The eigenvalues $\lambda_{n}\;$yield the modal frequencies, while the eigenvectors, $\Phi_{n},$ provide the mode shapes of structures. This is a general form of equation to compute modal frequencies and mode shapes. The relation between the eigenvalues $\lambda_{n}$ and $f_{n}$ is described as follows:

(6) $$\lambda_{n}=w_{n}^{2}$$

(7) $$w_{n}=\sqrt{\lambda_{n}}\;$$

(8) $$w_{n}={2\pi f}_{n\;}$$

where $w_{n}$ and $f_{n}$ represent the angular and linear natural frequencies, respectively.

The Lanczos methodology encompasses a set of Lanczos runs, with each run consisting of several iterations to refine the solution. The following spectral transformation is employed by Lanczos for each run.

(9) $$M(K-\sigma \;M{)}^{-1}\;M\Phi_{n}=ϴM\Phi_{n}$$

where σ and ϴ are the phase shift and eigenvalue, respectively. This transformation provides effective convergence to the expected eigenvalues. The eigenvectors of this equation (Equation (4)) and the spectral transform equation (Equation (9)) are the same, but the eigenvalues have the following relationship:

(10) $$\lambda_{n}=\frac{1}{ϴ}+\sigma \;$$

### 2.3. Frequency Response Analysis (FRA)

**Figure 4:**
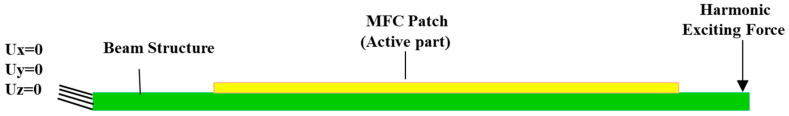
Schematic depiction of beam structure with MFC to assess frequency-dependent dynamic behavior.

The frequency-dependent dynamic response (amplitude spectrum) of each beam structure (PLAP, SCFR|PLAP, and CCFR|PLAP) with layers oriented at 0°|0° was assessed across a frequency spectrum and subjected to dynamic loading conditions. One side of the beam structure was firmly fixed, while the harmonic excitation force was applied to the free-end side, as exhibited in Figure 4.

The FRA primarily depends on the previously performed modal analysis, as described in Equation (9). Modal analysis determines the modal frequencies (${wʼ}_{n})$ and modal shapes of the beam structure, which are crucial for describing the dynamic behavior of the beam structure. FRA finds out the response of the structure subjected to harmonic force over a spectrum of frequencies. The displacement spectrum z(t) in response to the applied force can be stated as the cumulative total from each modal mode, as described in the Abaqus documentation [41]:

(11) $$z(t)=\sum_{n=1}^{\infty }\alpha_{n}\Phi_{n}$$

where $\alpha_{n}$ and $\Phi_{n}$ denote the modal amplitude and mode shape (mode coordinates) of nth mode, respectively. Where Equation (12) describes $\alpha_{n}$:

(12) $$\alpha_{n}={{(\Phi }_{n}}^{T}F_{0})/{(w}_{n}^{2}-w^{2}+2i\;{ʆ}_{n}w_{n}w)$$

In Equation (12), $F_{0}$, w and ${ʆ}_{n}$ denote the force exertion, excitation frequency, and damping ratio, respectively.

### 2.4. THz Spectroscopy

**Figure 5:**
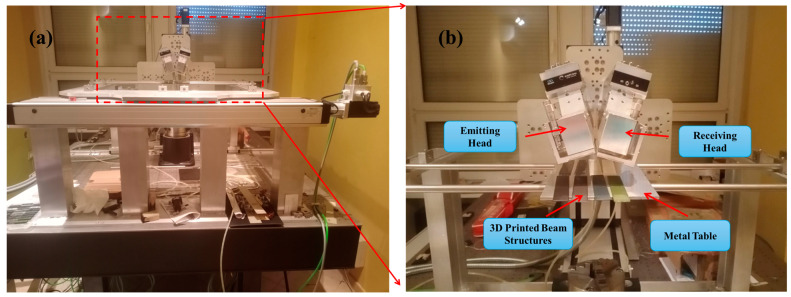
Non-destructive C-scanning of beam structures to identify the internal defects: (**a**) THz spectrometer setup for experiment and (**b**) illustration identifying internal defects.

The non-destructive C-scanning of beam structures was performed to identify internal defects using the THz spectrometer (TPSTM Spectra 300 THz Pulsed Imaging and Spectroscopy from TeraView), as presented in Figure 5a. This high-tech instrument employs terahertz (THz) radiation to analyze samples made from various materials. The unique characteristics of THz radiation enable it to penetrate samples, making it effective for non-destructive testing and imaging. The applicability of the NDT technique is primarily limited to the inspection of non-conductive materials, such as polymers [42] or glass fiber-reinforced polymers [43]. Its main advantage is the low power (below 1 µW [44]) generated by a THz wave that reduces the risk of destroying sensitive materials. THz waves are totally reflected by conductive materials. CCFR structures contain conductive carbon fibers reinforced in a non-conductive polymeric matrix. The applicability range of the NDT method for CCFR strongly depends on the carbon fiber content. For instance, CCFR laminates manufactured using infusion method have a high fiber content (typically 60–70% of total weight [45]). In such case, THz spectroscopy can be used to detect coatings (e.g., contaminations, thickness variation [46]) or surface damage (e.g., burn [47]). A comparative analysis of the influence of different carbon fiber alignments in CCFR laminates on THz wave penetration depth is thoroughly presented by authors [48]. In the analyzed CCFR|PLAP samples, the fiber content is significantly lower, approximately 18%. This lower amount of conductive material (carbon fiber) allows THz spectroscopy to inspect the whole structure.

The spectrometer facilitates the determination of sample parameters within the THz frequency range and is capable of scanning samples in either reflection or transmission mode. Its imaging capability allows for the measurement of samples up to 700 mm × 700 mm in size. For the investigation, each beam structure, with dimensions of 120 mm × 20 mm × 1.35 mm, was placed on a metal table and examined in reflection mode. The measuring heads were configured with a 22° angle between them, as presented in Figure 5b. Since THz radiation is significantly affected by moisture, the investigation was conducted using an air dryer to minimize moisture around the measuring heads and an air conditioner to maintain an ambient temperature of 20 °C. The scanning step was set to 0.2 mm in the xy plane, and THz signals were recorded with 10 averages.

### 2.5. Examination of Vibration Control with Influence of MFC

To analyze vibration damping, the dynamic response equation of the beam structure with MFC has been employed and is as follows:

(13) $$M\ddot{z}(t)+C\dot{z}(t)+Kz(t)=F_{ext}-F_{MFC}$$

Here, C represent the damping matrix, $\dot{z}(t)$ is the velocity vector, $F_{ext}$ denotes the external force on the beam to generate vibrations, and $F_{MFC}$ indicates the force provided by the MFC to damp vibration amplitude.

(14) $$\mathrm{Where}\;F_{ext}=F_{0}sin(wt)\mathrm{and}\;F_{MFC}=d_{31}AV_{0}sin(wt)$$

where A, $V_{0},$ and $d_{31}$ indicate the active area of the MFC, amplitude of harmonic signal provided to MFC, and piezoelectric coefficient, respectively.

The force ($F_{MFC}$) produced by the MFC operates in opposition to the external harmonic force and effectively reduces the vibration amplitude in the beam structure. The damping matrix C′, incorporated in the Equation (13), inherently encompasses the internal friction and other energy dissipative effects that are crucial to damp vibration amplitude in the beam structure.

## 3. Results and Discussions

To examine the dynamic characteristics of beam structures (PLAP, SCFR|PLAP, and CCFR|PLAP), modal characteristics’ assessment and FRA were carried out using the Abaqus. THz spectroscopy was carried out to identify defects in AM structures. Afterwards, vibration suppression in each beam was thoroughly studied and compared with the published experimental data [35], addressing the limitations of the numerical simulation.

### 3.1. Assessment of Modal Characteristics of Beam Structures

**Figure 6:**
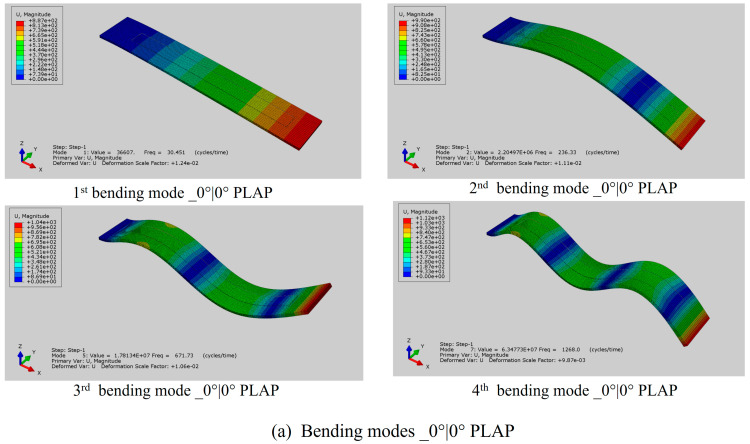

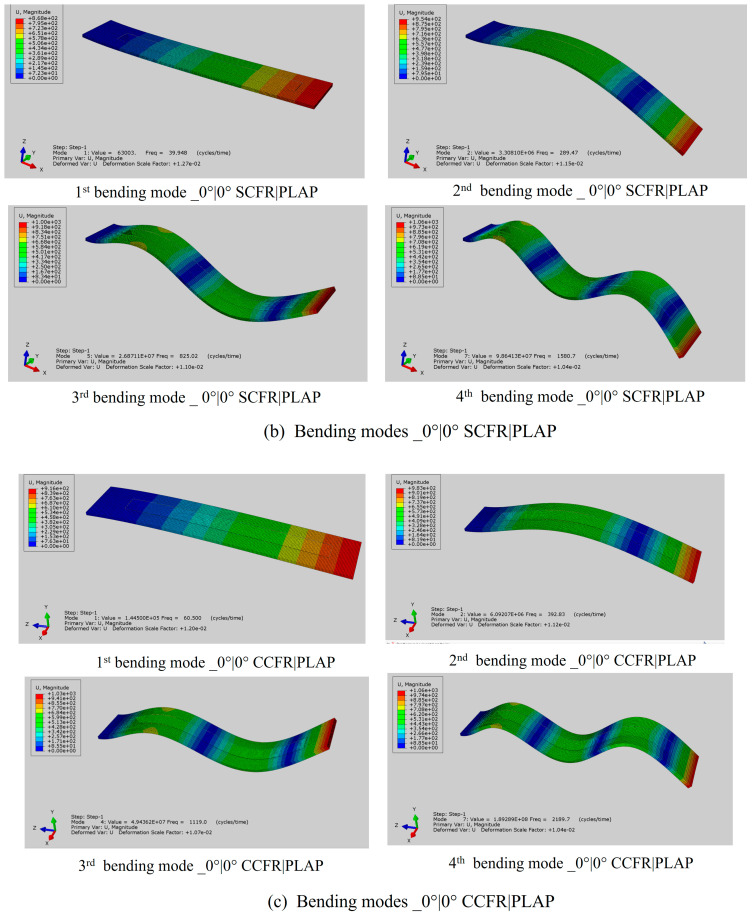
Initial four bending modes of MFC-integrated 0°|0°-oriented beam structures: (**a**) bending modes _0°|0° PLAP, (**b**) bending modes _0°|0° SCFR|PLAP, and (**c**) bending modes _0°|0° CCFR|PLAP.

In the preliminary phase of the numerical simulation investigation, the emphasis is on identifying the modal frequencies and modal bending mode shapes of numerical beam structures. By incorporating the MFC (M8507-P2) patch within the beams, each beam experiences a slight enhancement in mass and stiffness, leading to deviations in natural frequencies compared to beams without the MFC patch [24,35]. The natural frequencies of the first four bending mode shapes have been presented in Table 3, and it can be clearly observed that the natural frequencies of beams with the MFC are higher than those of beams without the MFC. The average computational time to calculate the natural frequencies of the numerical beam models was approximately 26 min. The initial four bending mode shapes of each numerical beam model are presented in Figure 6. The first natural frequencies of the numerical models were found to be 30.45 Hz for PLAP, 39.95 Hz for SCFR|PLAP, and 60.50 Hz for CCFR|PLAP. These numerical values closely align with the experimental frequencies of 30.00 Hz, 40.50 Hz, and 60.00 Hz, respectively, resulting in differences of only 1.50%, 1.35%, and 0.83% for PLAP, SCFR|PLAP, and CCFR|PLAP, respectively. The fourth natural frequencies of the numerical models were found to be 1268.00 Hz for PLAP, 1580.70 Hz for SCFR|PLAP, and 2189.70 Hz for CCFR|PLAP. The corresponding experimental values were 1207.50 Hz, 1546.00 Hz, and 2192.00 Hz, with differences of approximately 5.01%, 2.24%, and 0.10% for PLAP, SCFR|PLAP, and CCFR|PLAP, respectively. The second and third natural frequencies of the numerical models deviate within 10% from the experimental values.

**Figure 7:**
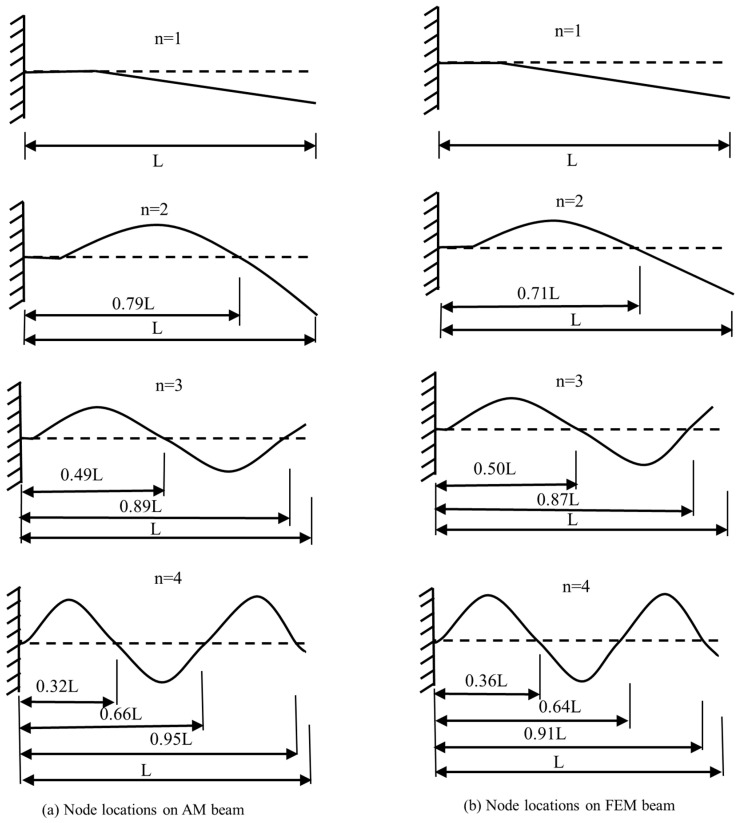
Node locations on beam structure integrated with MFC: (**a**) node locations on AM beam and (**b**) node locations on AM beam.

Several factors could contribute to discrepancies between the modal frequencies of the FEM and actual AM beam models. Firstly, the geometry of the AM beam could differ slightly from the idealized numerical model used in Abaqus. This difference might be due to internal irregularities such as defects that are not perfectly considered in the numerical model. Secondly, defining boundary conditions in the numerical model with simple assumptions could lead to inconsistencies with experimental results. For instance, in the numerical simulation, the beam structure is confirmedly fixed at one end, while in real conditions (experiments), there is a possibility that the beam was not firmly fixed, leading to a lack of consistency with the numerical model. To further investigate this, the node locations of the first four bending modes of one of the AM beams and its corresponding numerical model, specifically CCFR|PLAP with MFC, have been determined and presented in Figure 7. In the AM model, the node locations were identified using the 3D laser vibrometer, while in the numerical, the Abaqus platform was used. It can be clearly noted from Figure 7 that the beams vibrate very close to the fixed side, especially in the 2nd, 3rd, and 4th bending modes of both the AM and numerical models. There is a possibility that the AM beam was not firmly fixed, and displacement from the fixed end occurred during vibrating conditions.

### 3.2. Frequency Response Analysis (FRA) of Beam Structures

**Figure 8:**
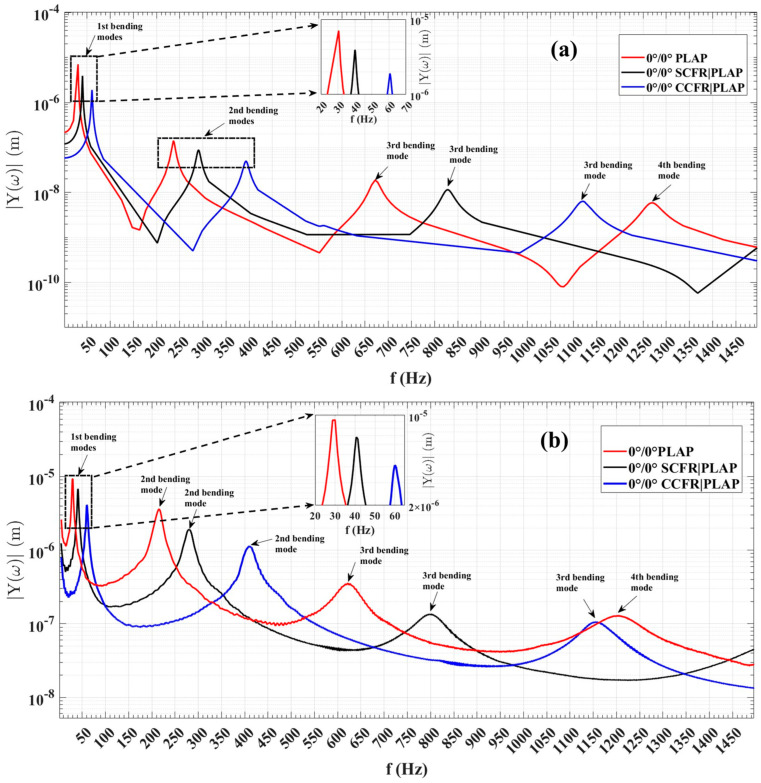
Frequency-dependent amplitude spectrum of 0°|0°-oriented beam structures: (**a**) numerical data and (**b**) experimental data.

In this section, a detailed discussion has been provided on the frequency-dependent dynamic behavior of the numerical beam models (PLAP, SCFR|PLAP, and CCFR|PLAP) integrated with the MFC under harmonic excitations. To assess the dynamic behavior, one side of numerical model was fixed, as depicted in Figure 4, while a sinusoidal force with an amplitude of ±1 × 10^−8^ N was applied to the other side. Figure 8 illustrates the amplitude spectrum of numerical and AM beam models at the first four resonant frequencies of bending modes. Table 4 presents the initial four bending mode numerical resonant frequencies of beam structures along with their amplitudes. The average computational time to compute the FRA response of beam structures was approximately 55 min.

These trends in numerical findings were confirmed with the early published experimental data [33]. The frequency-dependent dynamic response of the actual AM beam structures was measured by fixing one side of each beam structure in a fixture and applying a 100 V excitation signal to the MFC patch. The amplitude spectrum of each beam structure, illustrating the variation in vibration amplitudes with changing frequencies, was measured by a 3D laser vibrometer (PSV-W-500) manufactured by ‘Polytec GmbH, Germany’. Notably, the amplitude spectrum revealed (Figure 8) that vibration amplitudes peaked at the first resonant frequencies of the bending mode and decreased towards the fourth resonance frequencies in both numerical and experimental data. Although there was a considerable difference in the absolute values of vibration amplitudes between the numerical and experimental data, a consistent trend in amplitude variation over the resonant frequencies was observed in both sets of results.

Moreover, the resonant frequencies of FEM and AM beams were noted to be near each other, as reported in Table 3. Despite the discrepancy in the vibration amplitude values between the two datasets, there was consistency in the overall behavior of vibration amplitudes as frequency varied. For instance, in the numerical results, the vibration amplitudes at the first resonance frequency were observed to be the highest as follows: 7.0870 µm at 30.45 Hz for PLAP, 3.9240 µm at 40.00 Hz for SCFR|PLAP, and 1.8980 µm at 60.5 Hz for CCFR|PLAP. The corresponding experimental vibration amplitudes at the first resonance frequency were as follows: 9.295 µm at 30 Hz for PLAP, 6.831 µm at 40.5 Hz for SCFR|PLAP, and 4.139 µm at 60 Hz for CCFR|PLAP.

In the numerical results, the lowest vibration amplitudes were noted at the fourth resonant frequency: 0.0059 µm at 1268.00 Hz for PLAP, 0.0034 µm at 1585.10 Hz for SCFR|PLAP, and 0.0016 µm at 2189.70 Hz for CCFR|PLAP. The corresponding experimental values were observed to be 0.1290 µm at 1207.50 Hz for PLAP, 0.0490 µm at 1546.00 Hz for SCFR|PLAP, and 0.0220 µm at 2192.00 Hz for CCFR|PLAP.

Both the numerical and experimental results follow the same trend. Similarly, the variation in the vibration amplitudes of the numerical model at the second and third resonant frequencies follows the same trend observed in the experimental model, as illustrated in Figure 8.

Overall, the numerical approach effectively captures the dynamic behavior of each AM beam structure, as evidenced by the consistency of numerical results with the experimental results. However, the observed differences in absolute amplitude values may be attributed to the limitations in boundary conditions: in experimental case, the excitation force was provided by the MFC patch, while in numerical case, the force was provided at the free-end side of each beam structure.

### 3.3. Analysis of THz Spectroscopy Findings

The aim of utilizing THz spectroscopy was to analyze the beam structures of real samples that were modeled using the Abaqus CAE 2024 software. The simulations were performed under the assumption that the real structures were ideal, without voids or fiber alignment errors in any 3D-printed layer or across consecutive layers.

**Figure 9:**
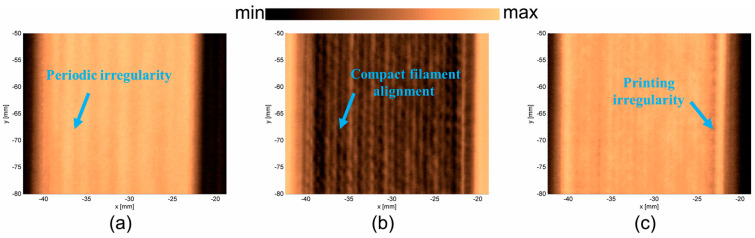
PLAP surfaces: (**a**) upper surface, (**b**) bottom surface, and (**c**) metal table.

C-scans were determined for three samples and presented in Figure 9a–c. In each case, three surfaces were determined: the upper surface of the sample, the bottom surface of the sample, and the image obtained from the metal table. In the case of PLAP (Figure 9), the image of the upper surface (Figure 9a) presents its periodic irregularity. The image of the bottom surface (Figure 9b) shows that the sample was manufactured from thin PLAP filament lines that were aligned so closely together they were not visible using the ‘naked eye’. Such internal structure influences the vibration properties of the material. The image obtained from the metal plate (Figure 9c) contains information about both the material’s periodic irregularity (probably due to the PLAP filaments in consecutive layers not being perfectly aligned) and the joint PLAP filaments creating the structure.

**Figure 10:**
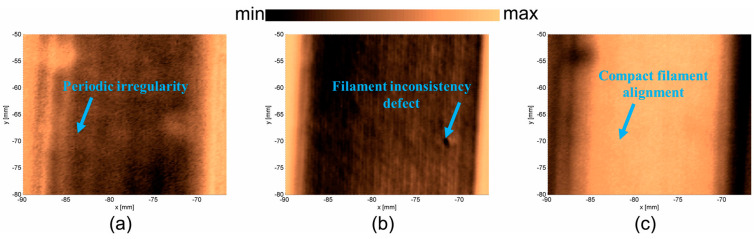
SCFR|PLAP surfaces: (**a**) upper surface, (**b**) bottom surface, and (**c**) metal table.

In the case of SCFR|PLAP (Figure 10), determining the upper surface was more challenging than in the PLAP case (pure polymer), probably due to the irregular distribution of conductive short carbon fibers in the SCFR|PLAP composite. Therefore, the image for the upper surface (Figure 10a) appears blurred. However, it is still possible to observe the periodicity of the sample surface. The image of the bottom surface (Figure 10b) indicates the characteristics of manufacturing method, revealing the sample was manufactured from thin parallel SCFR|PLAP filaments. The structure contains some discontinuations, observed as circular objects randomly distributed in the structure. This may be an effect of locally higher or lower amounts of short carbon fibers in the SCFR|PLAP-printed filaments. Such inequality should not considerably influence the vibrational properties of the material. However, differences in the mechanical properties of carbon fiber and polymer could result in such areas to be an origin of structural damage. The image determined from the metal table (Figure 10c) indicates that, when considering the whole thickness of the sample, the short carbon fibers are equally distributed.

**Figure 11:**
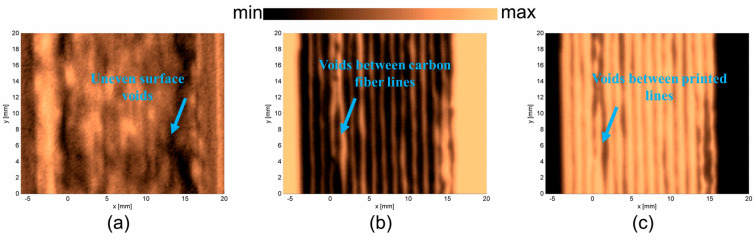
CCFR|PLAP surfaces: (**a**) upper surface, (**b**) bottom surface, and (**c**) metal plate.

In the case of CCFR |PLAP (Figure 11), the upper surface image (Figure 11a) shows that it is not perfectly flat, and the carbon fibers are not perfectly aligned. The areas with darker colors can be described as voids and regions with a higher amount of PLAP (pure polymer). The images of the bottom surface (Figure 11b) and metal table (Figure 11c) confirm the presence of voids. These are visible as irregular light lines (Figure 11b) between the carbon fibers and as irregular dark lines (Figure 11c) between reflections from the metal table. Such voids and misalignments of carbon fibers influence the vibrational parameters of the analyzed CCFR |PLAP structure, indicating potential origins of structural damage.

### 3.4. Analysis of Vibration Amplitude Control in Beam Structures

**Figure 12:**
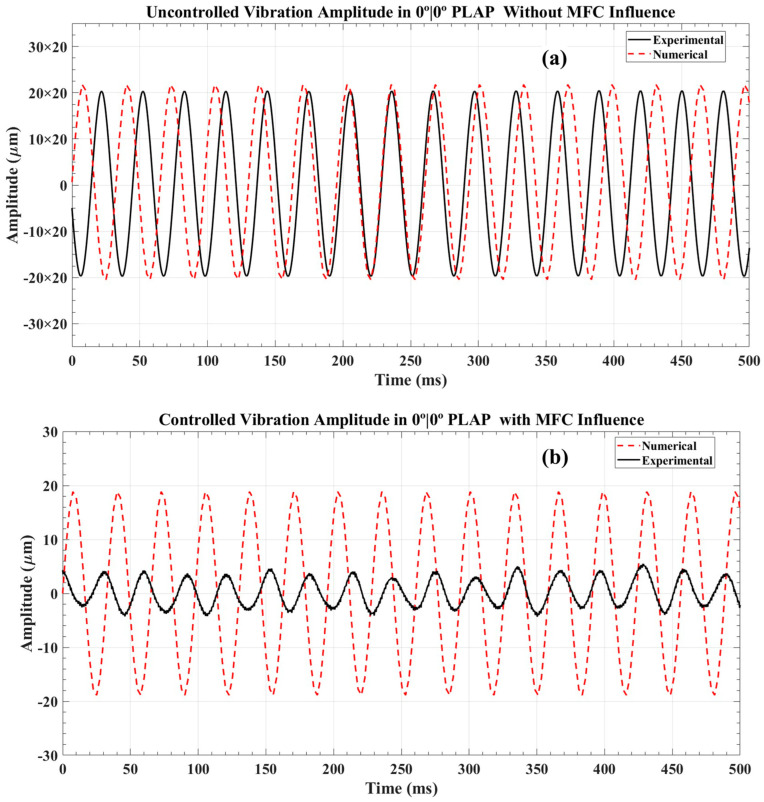
Vibration amplitude analysis in 0°|0° PLAP beam structure: (**a**) uncontrolled vibration amplitude and (**b**) controlled vibration amplitude.

This analysis provides a comprehensive discussion on vibration amplitude control in numerically modeled beam structures, comparing and validating the results obtained with experimental data. The damping characteristics of the numerical beam structures are adjusted to ensure that the numerical and experimental absolute uncontrolled vibration amplitudes are comparable. The average computational time to determine the controlled vibration amplitude in beam structures was around 97 min. The PLAP numerically modeled beam exhibited the highest uncontrolled vibration amplitude ranging from +435 µm to −408 µm when subjected to a harmonic force at its first resonant frequency of 30.45 Hz, as depicted in Figure 12a. To obtain an uncontrolled amplitude approximately identical to the experimental results, a specific force value for each beam was determined using the following expression [49]:

(15) $$F_{ext}=3YID/L^{3}$$

where $F_{ext}$**,** Y, I, D, and L indicate the load at the free end, Young’s Modulus, moment of inertia related to the cross-section, maximum peak-to-peak deflection, and length of each beam, respectively.

By applying a counter force through the MFC at the same resonant frequency (30.45 Hz), the maximum controlled vibration amplitude was observed to be approximately ±19 µm (see Figure 12b). This represents an overall reduction in controlled vibration amplitude of more than 22 times compared to the uncontrolled scenario. In the experimental scenario, the AM beam structure was stimulated at its first resonant frequency (30 Hz) with an electromagnetic shaker, resulting in an uncontrolled vibration amplitude of approximately ±410 µm. Applying a counterforce with the MFC at the same resonant frequency (30 Hz), the controlled amplitude was reduced to around ±5 µm. This demonstrates an overall reduction in controlled vibration amplitude of around 80 times.

**Figure 13:**
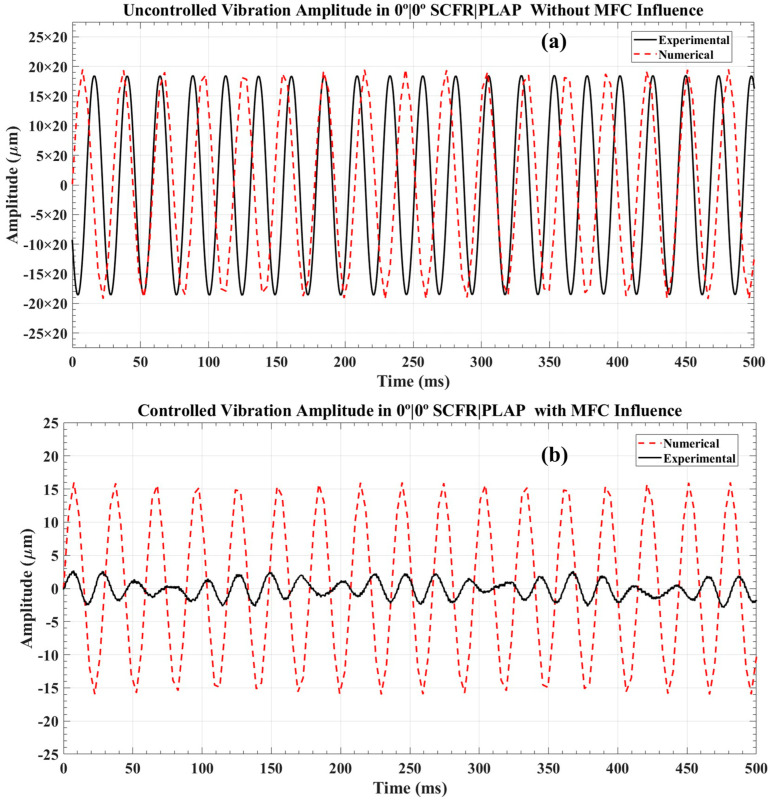
Vibration amplitude analysis in 0°|0° SCFR|PLAP beam structure: (**a**) uncontrolled vibration amplitude and (**b**) controlled vibration amplitude.

For the SCFR|PLAP numerical model, the uncontrolled highest vibration amplitude was observed around ±395 µm when subjected to a harmonic force near its first resonant frequency of 40 Hz, as depicted in Figure 13a. In experiments with the AM model, the uncontrolled amplitude was noted around ±370 µm at its first resonant frequency of 40.5 Hz. Upon applying a counterforce through the MFC, the controlled numerical and experimental vibration amplitudes were observed around ±16 µm and ±3 µm, respectively, as presented in Figure 13b. This reflects an overall reduction in controlled vibration amplitudes of approximately 25 times and 110 times, respectively.

**Figure 14:**
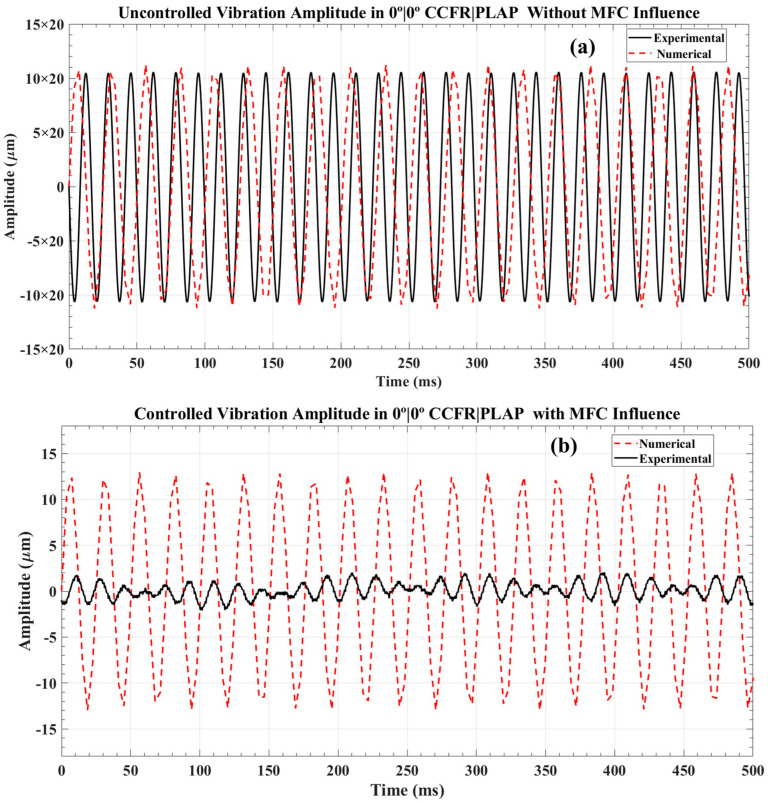
Vibration amplitude analysis in 0°|0° CCFR|PLAP beam structure: (**a**) uncontrolled vibration amplitude and (**b**) controlled vibration amplitude.

In both the numerical and AM models of the CCFR|PLAP, the numerical model exhibited uncontrolled amplitudes ranging from +220 µm to −224 µm, while the AM model showed an approximate amplitude of ±210 µm, as illustrated in Figure 14a. These measurements were taken at their respective first resonant frequencies, approximately 60.5 Hz for the numerical model and 60 Hz for the AM model. After the MFC applied a counterforce, the numerical and AM beams exhibited controlled amplitudes of ±13 µm and ±1.7 µm, respectively, as demonstrated in Figure 14b. This represents an overall reduction in controlled amplitudes of approximately 31 times for the numerical model and 120 times for the AM model.

The results demonstrate significant changes in the reduction ratios for PLAP, SCFR|PLAP, and CCFR|PLAP in experimental cases, with reductions of 80 times, 110 times, and 120 times, respectively. In contrast, numerical models exhibited reductions of 22 times, 25 times, and 31 times, respectively. However, the peak-to-peak controlled vibration amplitudes remain relatively consistent, such as ±5 µm, ±3 µm, and ±1.7 µm for PLAP, SCFR|PLAP, and CCFR|PLAP in experimental scenarios, and ±19 µm, ±16 µm, and ±13 µm in numerical scenarios, respectively. It indicates consistent effectiveness in controlling vibration amplitudes in both numerical and experimental scenarios.

The disparity in controlled vibration amplitudes between the numerical and experimental results is influenced by the inherent limitations of the numerical models, which do not fully capture the complexities of real experimental conditions. Numerical simulations typically rely on generalized assumptions and may not properly account for material imperfections such as voids, porosity, or any irregularities, which are present in the actual model. Additionally, environmental factors and intricate damping characteristics resulting from the fixture used to secure one side of the beam during experiments are not fully considered in the numerical simulation.

The numerical findings provide valuable insights for estimating trends related to vibration control in AM beam structures. However, the experimental data are crucial for improving and optimizing vibration amplitude control methodologies to assure their efficacy in real-life applications. Despite the disparity in controlled vibration amplitudes between numerical and experimental models, the trend of reduction factor in both scenarios is similar. It increases from PLAP to SCFR|PLAP and then continues with CCFR|PLAP.

## 4. Conclusions

In this article, the numerical models of 0°|0° PLA, 0°|0° SCFR|PLA, and 0°|0° CCFR|PLA beam structures integrated with the MFC have been investigated to study their dynamic characteristics and control vibration amplitude under kinematically stimulated conditions. The numerical results are comprehensively compared and validated against previously published experimental results. In response to the findings from the results, the subsequent conclusions are formulated.

1. The 1st resonant frequencies were found to rise from 30.45 Hz for PLAP, to 39.95 Hz for SCFR|PLAP, and then to 60.5 Hz for CCFR|PLAP. The numerical values align with experimental values of 30 Hz, 40.5 Hz, and 60 Hz, with discrepancies of 1.50%, 1.35%, and 0.83%, respectively.
2. Numerical analysis of the frequency-dependent amplitudes of the initial four bending modes exhibited that the 1st resonant frequency had the highest amplitudes, while the lowest amplitudes were observed at the 4th resonant frequencies. A similar trend in frequency-dependent amplitudes was observed in the experimental scenario.
3. THz spectroscopy was performed to identify voids or misalignments in the real AM samples. The PLAP revealed periodic irregularities, SCFR|PLA showed random discontinuities, and CCFR|PLA showed a significant number of voids and misalignments in the continuous carbon fiber.
4. The numerically models of PLAP, SCFR|PLAP, and CCFR|PLAP were externally stimulated at their respective 1st resonant frequencies, revealing uncontrolled vibration amplitudes of +435 µm to −408 µm, ±395 µm, and +220 µm to −224 µm, respectively. In the experimental case, uncontrolled vibration amplitudes were ±400 µm, ±370 µm, and ±210 µm.
5. After applying a counterforce with the MFC, the controlled vibration amplitudes in the numerical models for PLAP, SCFR|PLAP, and CCFR|PLAP were approximately ±19 µm, ±16 µm, and ±13 µm, respectively. Experimentally, the controlled vibration amplitudes were observed to be around ±5 µm, ±3 µm, and ±1.7 µm for the respective models. Both numerical and experimental results exhibited the same trend in decreasing amplitude from PLAP to SCFR|PLAP and finally to CCFR|PLAP.
6. Numerical simulation provides valuable insights for estimating trends in controlling vibration amplitudes. However, experimental data are crucial for improving and optimizing vibration amplitude reduction methodologies to ensure their efficacy in real-life applications.

## Figures and Tables

**Table 1:** Mechanical parameters of PLAP, SCFR|PLAP, and CCF [22,36,37,38].

Parameters	PLAP	PLAP|SCF	CCF
Young’s Modulus (Y)	(2636 ± 330) × 10^6^ Pa	5143 × 10^6^ Pa	230 × 10^9^ Pa
Poisson’s Ratio (υ)	0.36	0.4	0.2
Tensile Strength	(46.6 ± 0.9) × 10^6^ Pa	76 × 10^6^ Pa	3530 × 10^6^ Pa
Density	1.17 to 1.24 g/cm^3^	1.35 g/cm^3^	1.76 g/cm^3^

**Table 2:** Material Parameters of MFC (M8507-P2) [20,22,39,40].

Parameters	Values
Young’s Modulus (Y)	Y_1_ = 30.336 × 10^9^ Pa
	Y_2_ = Y_3_ = 15.857 × 10^9^ Pa
Shear Modulus	G_12_ = G_13_ = G_23_ = 5.515 × 10^9^ Pa
Poisson’s Ratio (υ)	υ_12_ = υ_13_ = 0.31
	υ_23_ = 0.438
Density	5.44 g/cm^3^
Piezoelectric Coefficient	d_31_ = −170 pm/V
	d_32_ = −100 pm/V

**Table 3:** Natural frequencies of 0°|0°-oriented beam structures with and without MFC: experiments and numerical simulation results.

Bending Modes	0°|0° PLAP			0°|0° SCFR|PLAP			0°|0° CCFR|PLAP		
	Experiment with MFC (Hz)	Numerical Without MFC (Hz)	Numerical with MFC (Hz)	Experiment with MFC (Hz)	Numerical Without MFC (Hz)	Numerical with MFC (Hz)	Experiment with MFC (Hz)	Numerical Without MFC (Hz)	Numerical with MFC (Hz)
1st	30.00	26.89	30.45	40.50	34.70	39.95	60.00	61.47	60.5
2nd	215.00	168.23	236.33	280.50	216.96	289.47	410.00	384.71	392.83
3rd	626.50	471.76	671.73	802.00	608.53	825.02	1153.00	1076.10	1119.00
4th	1207.50	927.35	1268.00	1546.00	1196.50	1580.70	2192.00	2106.20	2189.70

**Table 4:** Numerical natural frequencies of 0°|0°-oriented beam structures with amplitudes.

Bending Modes	0°|0° PLAP		0°|0° SCFR|PLAP		0°|0° CCFR|PLAP	
	Numerical Frequency (Hz)	Amplitude (µm)	Numerical Frequency (Hz)	Amplitude (µm)	Numerical Frequency (Hz)	Amplitude (µm)
1st	30.45	7.0870	40.00	3.9240	60.50	1.898
2nd	236.33	0.1420	290.42	0.0874	392.83	0.0496
3rd	671.73	0.0189	827.70	0.0115	1119.00	0.00636
4th	1268.00	0.0059	1585.10	0.0034	2189.70	0.0016

## Data Availability

The original contributions presented in the study are included in the article, further inquiries can be directed to the corresponding authors.

## Abbreviations

**Acronym**	**Full Term**
MFC	Macro fiber composite
PLAP	Polylactic acid polymer
SCFR|PLAP	Short carbon fiber reinforced in PLAP
CCFR|PLAP	Continuous carbon fiber reinforced in PLAP
CGFR|PLAP	Continuous glass fiber reinforced in PLAP
FRA	Frequency response analysis
NDT	Non-destructive testing
BM	Bending mode
AM	Additively manufactured
FEM	Finite element modeling
